# Revisiting the COVID-19 fatality rate and altitude association through a comprehensive analysis

**DOI:** 10.1038/s41598-022-21787-z

**Published:** 2022-10-27

**Authors:** Carson Bridgman, Jacob Gerken, Joshua Vincent, Amanda E. Brooks, Isain Zapata

**Affiliations:** 1 Department of Biomedical Sciences, Rocky Vista University, College of Osteopathic Medicine, 8401 S. Chambers Rd., Parker, CO 80134 USA; 2 Department of Research and Scholarly Activity, Rocky Vista University, College of Osteopathic Medicine, Ivins, UT 84738 USA

**Keywords:** Diseases, Health care, Risk factors

## Abstract

The emergence of COVID-19 virus has led to a pandemic with staggering morbidity and mortality. There is evidence showing that pre-existing conditions and environmental factors are associated with worse COVID-19 outcomes. Among these conditions, altitude is of particular interest. Altitude has been shown to influence the morbidity and mortality of multiple chronic pathologies such as cardiovascular disease, chronic obstructive pulmonary disease and lung cancer. COVID-19 fatality rate has been associated with as altitude as well, but findings are disputed. Therefore, we revisit this assessment with a comprehensive analysis of the relationship between COVID-19 fatality rates and altitude for the Mountain region of the United States while considering the effect of additional comorbidities and sociodemographic factors. A Generalized Additive Model (GAM) approach using one year of county data adjusted by population density was performed to evaluate associations within states and for the whole region. Our analysis revealed a consistent effect where COVID-19 case-fatality rate is decreased with higher altitude, even when controlling for pre-existing conditions and certain demographic variables. In summary, the work presented provides evidence that suggests that the protective effects of high altitude are likely to be influenced by physiologic factors but demographic trends that are associated with life at high altitude must also be considered.

## Introduction

The COVID-19 virus has caused a worldwide pandemic with progressive cases and fatalities. Not only has the United States been hard hit by the pandemic (as of this publication it was reporting the highest number of deaths and cases on a per country basis), but it is also leading many research efforts to combat the virus^1^. A key component of many of these research studies focused on evaluating relationships between COVID-19 mortality and numerous variables such as pre-existing conditions and age^2^. This renewed interest in public health and epidemiological studies has led researchers to identify several socioeconomic factors that could be correlated to prevalence and fatality of COVID-19. Specifically, the socioeconomic variables that had the highest risk ratios for both infection and fatality due to COVID-19 were low education and black ethnicity even when compared to unemployment, income, lack of health insurance, COPD, diabetes, and obesity^3^. Considering a country’s social and demographic attributes is essential when modeling factors influencing the pandemic. A country’s current health expenditure as a function of GDP^4^ and income inequality within a country^5^ are also linked to COVID-19 spread and vulnerability. While many socioeconomic factors have been investigated to frame evidence-based recommendations in our fight against COVID-19, one factor that has been recently, but only superficially analyzed is altitude. An observational and modeling study detected an association between higher altitude with decreased infection, ultimately recommending a deeper analysis of this idea^6^. Furthermore, two additional studies have been conducted supporting the idea that increased altitude may decrease the pathogenesis of COVID-19^6–8^.

The link between altitude and pathology is not a new revelation. Numerous studies have looked into the effect of moderate altitude gain with respect to fatality in other pathologies, including lowering of overall mortality in cardiovascular and cerebrovascular diseases^9^. Also, higher altitudes have been identified as a risk factor for undiagnosed COPD^10^ and lung cancer^11^. These generalized associations suggest that altitude is likely to have some effect on COVID-19 etiology. Temperature is another geographic variable commonly evaluated with altitude that was initially thought to influence the spread of COVID-19^12^. The results concerning temperature are mixed, with a pair of studies^12,13^ showing higher temperatures slowing the contagion rates, and others showing temperature having a weak correlation^5,14^ or varied effect of COVID-19 spread depending on altitude^15^. Etiological factors can be related to the physiological adaptation often seen in response to chronic exposure to higher altitudes. Such exposure may result in increased lung diffusion capacity, increased capillary blood volume, decreased blood pressure, polycythemia, and increased O_2_ carrying capacity of blood^16^. The positive effects of altitude on lung function could be an indication of alterations in the expression level of Angiotensin-converting enzyme 2 (ACE2), which is the primary route of entry for the COVID-19 virus into cells. In addition, it seems to play a pivotal role in acute lung injury and other organ damage^17^. Therefore, it is a reasonable hypothesis that these physiological responses could positively impact the defense against COVID-19, ultimately reducing fatality rates in moderate-high altitude populations. There have been studies early on in the pandemic evaluating this altitude’s effect on fatality rates with an active COVID-19 infection, however these studies did not consider any sociodemographic covariates^18,19^, or were done very early in the pandemic without an equal distribution of active COVID-19 infections^20–22^.

The association of higher altitude to decreased COVID-19 fatality rates has not been evaluated in the context of comorbidity risk factors (e.g., COPD, CHD, obesity, and smoking) or socioeconomic risk factors (e.g., gender, age, ethnicity, education, and income), which are known to be associated to COVID-19 fatalities. We revisit this premise by presenting this study that evaluates the effect of altitude on COVID-19 fatality rates while simultaneously considering confounding effects of comorbidities and demographic risk factors. The study was performed using county aggregated metrics for states located in the Mountain region of the United States, this region includes approximately 25 million people which is close to 8% on the total population in the country. We focus on this region as it provides us with the required dynamic representation of altitude as all eight states in this region have a wide range of altitude. Our study provides further information that connects COVID-19 to etiological factors, leading to advanced management strategies to reduce fatality rates and improve outcomes.

## Methods

### Study design

**Figure 1:**
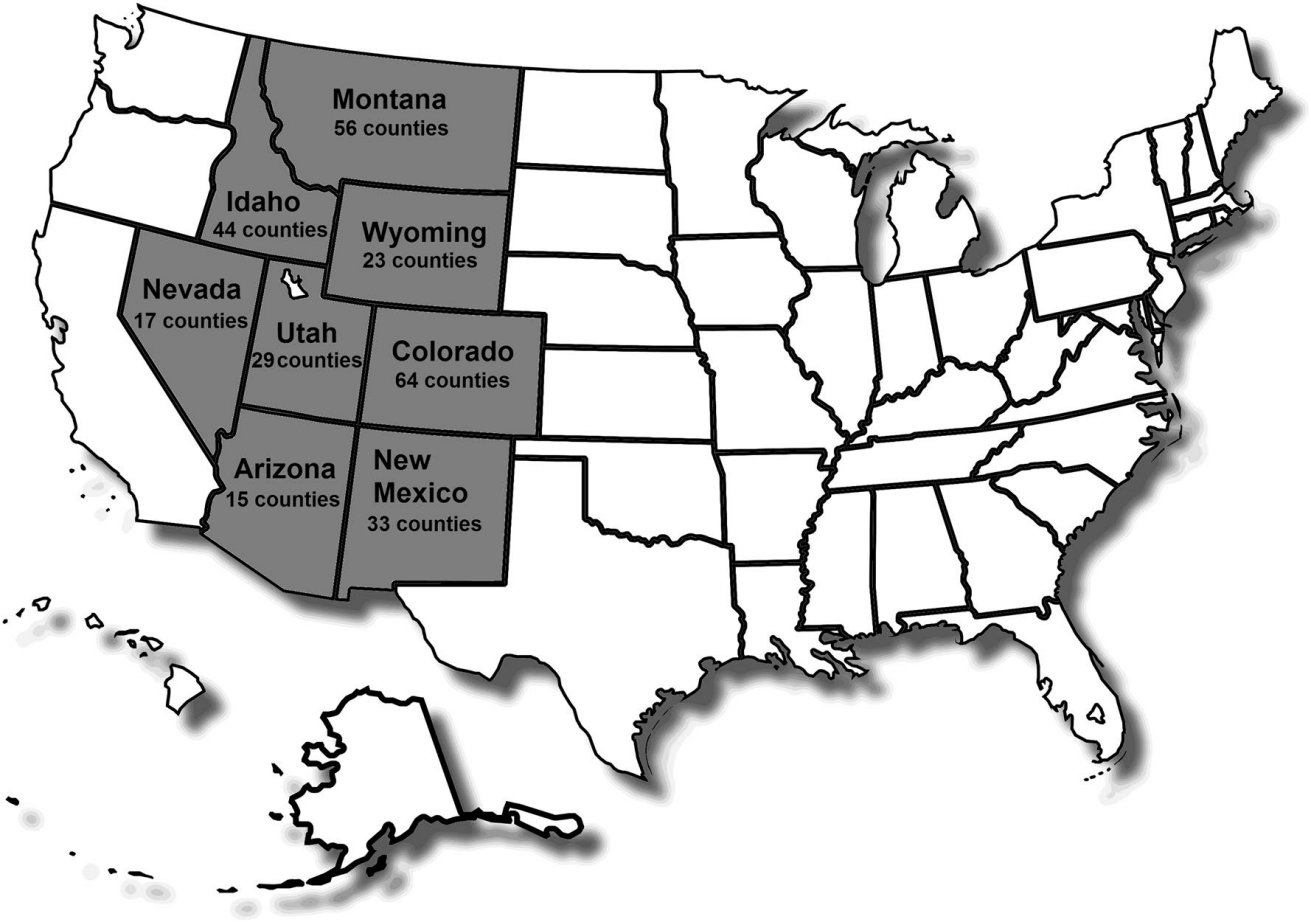
States included in this study. These states are defined as the Mountain State Region as recognized by the United States Census Bureau.

The study was designed to focus on state and regional trends between associations of COVID-19 fatalities over one year (March 2020 to March 2021) to altitude while considering the effect of comorbidity rates and demographic factors. To achieve this, a statistical modeling approach that used county-wide aggregated data to elucidate associations was developed and adjusted to population density. The study focused on the Mountain region of the western United States; this region contains the US Rocky Mountains, which is the major mountain range in western North America and is a sub region recognized by the US Census Bureau. All states included in the Mountain region are presented in Fig. 1. All data used in this study was obtained from previously published sources or public databases without the use of any specific identified data. All methods were performed in accordance with relevant guidelines and regulations.

### Altitude, comorbidity, demographics, and COVID-19 fatality data

County altitude data was obtained from a previously published dataset evaluating lung cancer incidence^11^. Altitude data from that study was a population-weighted mean average, which was calculated for all counties in the United States by subdividing each county into a census block, then determining the weighted average of the block's altitude. This method is argued by the authors to be a better estimate of an individual’s exposure because it accounts for the geographic distribution of population density. The COVID-19 fatality rate for each individual county was obtained from each state’s official COVID-19 website (resources available as Supplementary File 1). The data in this study records all COVID-19 cases and deaths reported on each of the state’s official websites as of March 14th, 2021. Our study used only official reported values and did not attempt to recalculate or considered possible COVID-19 fatalities not officially defined. Altitude and COVID-19 county data averaged by state and Mountain region are presented in Table 1.

**Table 1:** Mean and standard deviations for county altitude and COVID-19 cases, deaths and case fatality by state and by mountain region. Fatality rate is expressed as the fatal COVID-19 cases/Total number of cases for each county.

Region	Counties and altitude					COVID-19 county averages		
	Total number of counties	Average county population (people)	Average county altitude (km)	County at lowest altitude	County at highest altitude	County COVID-19 cases	County COVID-19 deaths	COVID-19 case fatality
**Arizona**								
Mean	15	459,415	1.068	Yuma, 0.060 km	Apache, 1.971 km	55,242	1094	0.0243
Std Dev		1,083,552	0.607			130,873	2354	0.0069
**Colorado**								
Mean	64	85,775	2.061	Sedgwick, 1.090 km	San Juan, 3.473 km	6332	90	0.0144
Std Dev		175,111	0.651			13,044	187	0.0113
**Idaho**								
Mean	44	38,325	1.301	Nez Perce, 0.427 km	Custer, 2.306 km	3978	43	0.0117
Std Dev		75,774	0.465			8305	83	0.0065
**Montana**								
Mean	56	18,308	1.151	Richland, 0.624 km	Madison, 1.858 km	1816	24	0.0200
Std Dev		32,054	0.340			3310	40	0.0153
**New Mexico**								
Mean	33	62,121	1.683	Eddy, 1.011 km	Mora, 2.534 km	5702	117	0.0239
Std Dev		119,605	0.409			10,059	185	0.0148
**Nevada**								
Mean	17	172,346	1.504	Clark, 0.655 km	White Pine, 2.021 km	17,590	301	0.0163
Std Dev		521,606	0.326			55,944	967	0.0102
**Utah**								
Mean	29	105,133	1.765	Washington, 1.005 km	Wayne, 2.383 km	12,972	70	0.0074
Std Dev		232,874	0.336			30,574	159	0.0053
**Wyoming**								
Mean	23	24,649	1.687	Sheridan, 1.284 km	Sublette, 2.448 km	2398	30	0.0139
Std Dev		23,656	0.350			2307	34	0.0073
**Mountain region**								
Mean	281	84,299	1.569	Yuma, AZ, 0.060 km	San Juan, CO, 3.473 km	8645	132	0.0161
Std Dev		314,553	0.581			36,511	635	0.0121

Comorbidity data by county for obesity, chronic obstructive pulmonary disease (COPD), coronary heart disease (CHD), asthmas, diabetes, smoking, and chronic kidney disease (CKD) was obtained from the Population Level Analysis and Community Estimates (PLACES) project which is sponsored by the Centers for Disease Control and Prevention^23^. Comorbidities were generated using the results from the 2018 Behavioral Risk Factor Surveillance System (BRFSS). Demographic data used for this study was obtained from the American Community Survey (ACS) 2019, 5-year subject tables from the United States Census Bureau^24^. Comorbidity and demographic factor data averaged by state and Mountain region in this study are presented in Supplementary Table 1.

### Statistical analysis

This is study used Nonparametric Regression models applied through a Generalized Additive Models (GAMs) approach. GAMs were selected because they can address deviations from normality, which are a limitation of Generalized Linear Models (GLMs)^25,26^. These models use non parametric smoothing splines that a linked to the predictor evaluated^27^. The models evaluate associations between COVID-19 fatality rate, which was set as the dependent variable, to altitude, comorbidity, and demographic factor effects, which were set as independent variables. Each of the evaluations was performed by state and all models are adjusted to population density. Regional analysis (as a meta-analysis) was done independently for each variable. All models used the individual county as the experimental unit. Dependent variables were introduced into the model as non-parametric smoothing splines with 3 degrees of freedom. GAM used were as follows:

$${{COVID}}{\text{-}} {19}\;{{Fatality}}\;{{rate}}_{{{\text{ijkl}}}} = \beta_{0} + s_{i} \;(Population\;density_{i} ) + s_{j} \;(Altitude_{j} ) + s_{k} \;(Covariates_{k} ) + \varepsilon_{ijkl}$$

where *COVID-19 Fatality rate*_*ijkl*_ is the dependent variable measured on the individual county. *β*_*0*_ is the intercept. *s*_*i*_, *s*_*j*_ and *s*_*k*_ are smoothing functions estimated in a non-parametric fashion. *Population density*_*i*_, *Altitude*_*j*_, and *Covariates*_*k*_ are the specific population density, altitude and covariates (sociodemographic and comorbidities) at the individual county. *ε*_*ijkl*_ is the random error inherent to each measurement (residuals) which is assumed to be independent of other observations. In summary, the generalized GAM equation is very similar to the GLM but adds spline functions that models internally each independent parameter included in the model. All models assumed Gaussian distribution for the residuals, which were corroborated through preliminary residual plots. All non-parametric modeling was performed using PROC GAM in SAS/STAT v.9.4 (SAS Inc., Cary, NC). Modeling effects were estimated as Risk Ratios with 95% confidence intervals; effect directions were determined by the estimate coefficient sign. A negative coefficient would reduce the fatality rate while a positive one would increase the rate. Standardization of coefficients was done using a normally distributed z-score transformation. Descriptive statistics were calculated by state and by region using PROC MEANS in SAS/STAT. All significant associations presented considered a two tailed test. Bonferroni level significance was declared per family of tests which is disclosed in each figure. The code and data used in the analysis is available as supplemental material.

## Results

**Figure 2:**
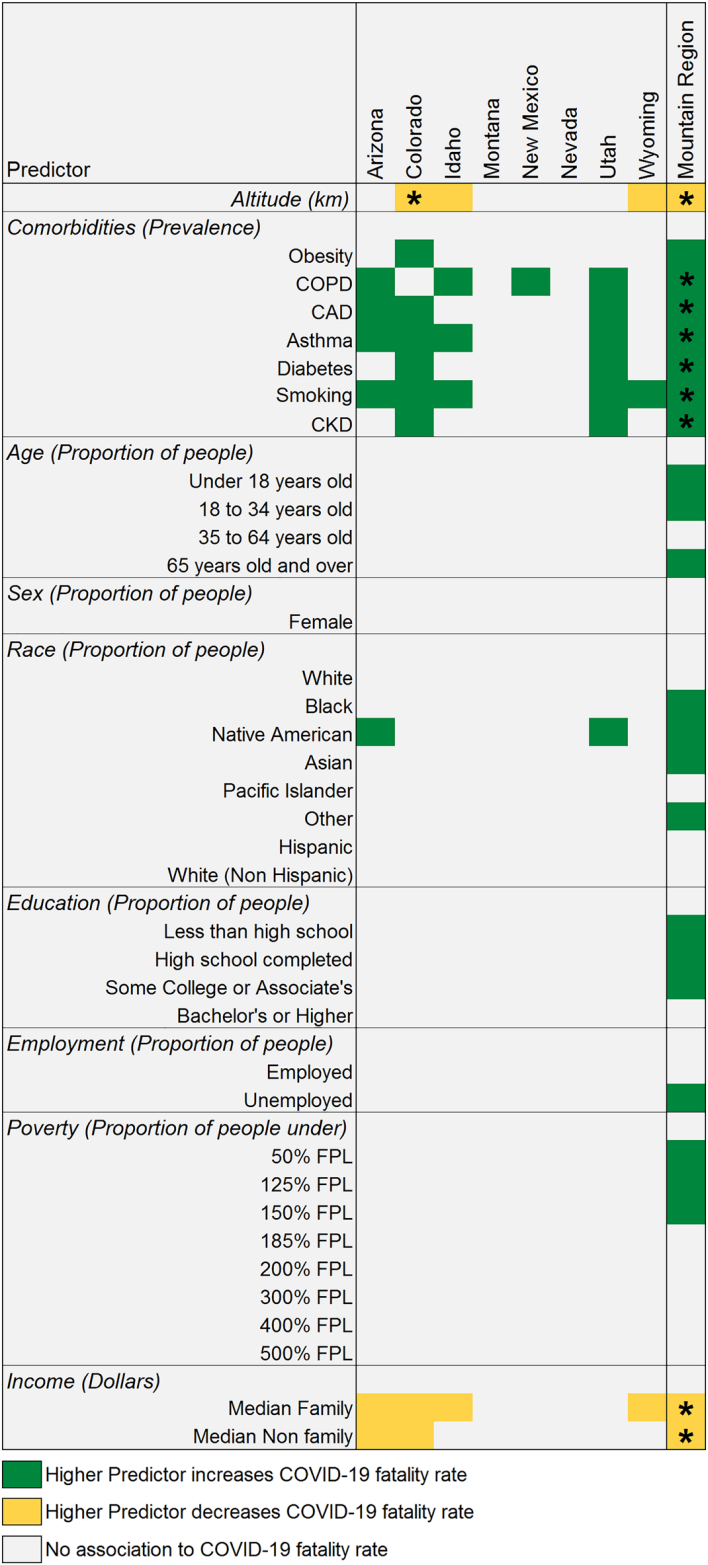
COVID-19 fatality rate association to county-wide comorbidities and demographic factors adjusted by population density. Only those associations that were detected as significant (P ≤ 0.05) are shown. Asterisk (*) indicates significance of a specific predictor at a Bonferroni threshold (37 tests). Mountain region corresponds to all states evaluated as a meta-analysis.

County aggregated data analyzed with a Nonparametric Regression approach though Generalized Additive Models adjusted by population density was used to evaluate the association between COVID-19 fatality rate to altitude, comorbidities, and demographic factors. The outcomes of this modeling approach are presented in Fig. 2. Between the states within the Mountain region, some association with altitude and fatality rate (Colorado, Idaho, and Wyoming) was observed, demonstrating higher altitude lowering the COVID-19 fatality rate. This association was also observed for the whole Mountain region when analyzed as a meta-analysis. Higher comorbidity rates were associated with higher COVID-19 fatality rates within states (except for Montana and Nevada) and for the Mountain region collectively. Associations significant to a Bonferroni level were only detected for the Mountain region. The states of Arizona, Colorado, Idaho, and Wyoming displayed an association to income; in all cases a higher median income was associated with lower COVID-19 fatality rate. This trend was also observed for the Mountain region significant at a Bonferroni confidence level. Associations to age, education, employment status and poverty were only detected for the Mountain region. The effect of race was detected for the states of Arizona and Utah and for the whole Mountain region.

**Figure 3:**
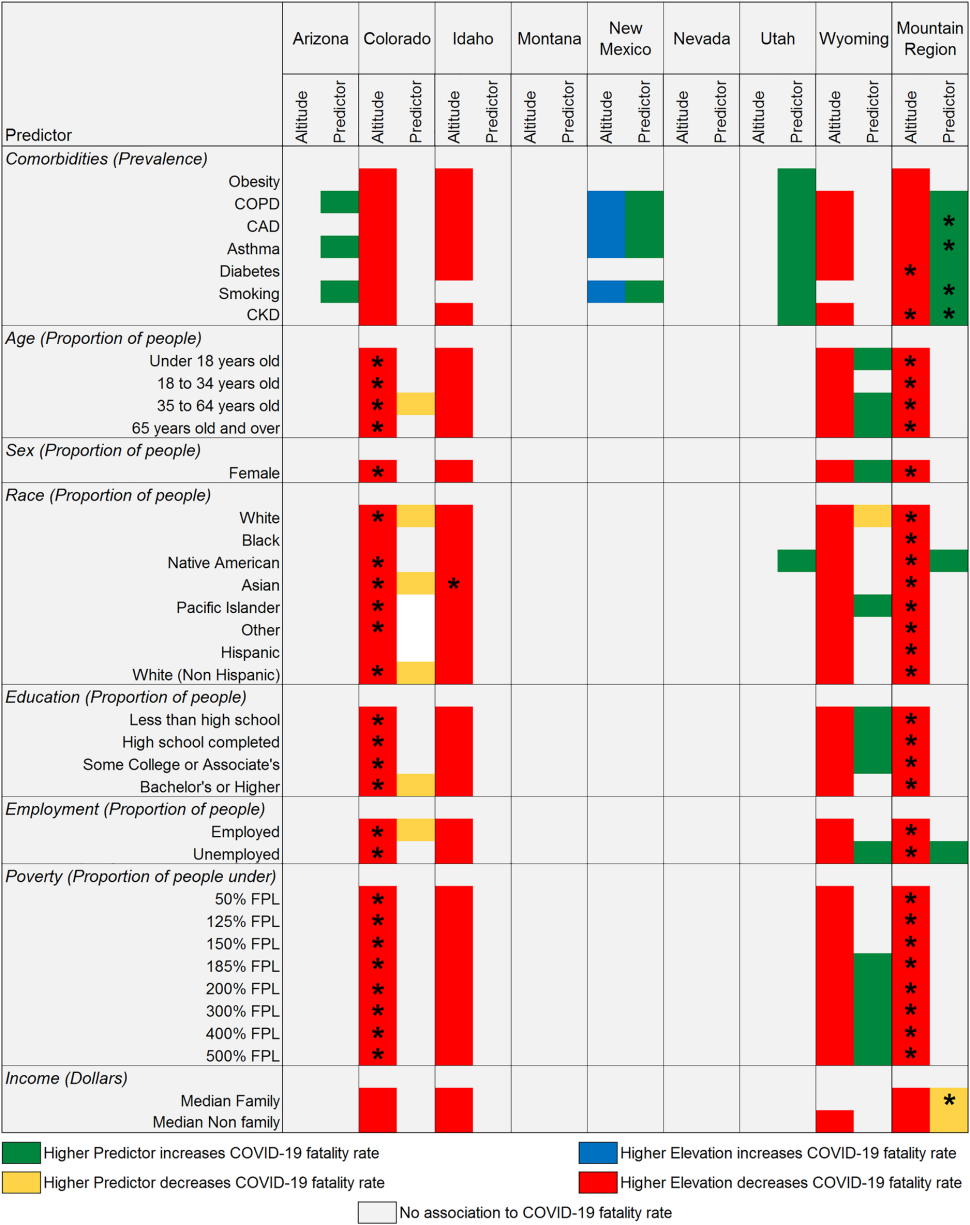
COVID-19 fatality rate association to county-wide comorbidities and demographic factors while simultaneously evaluating mean county altitude associations adjusted by population density. Only those associations that were detected as significant (P ≤ 0.05) are shown. Asterisk (*) indicates significance of a specific predictor at a Bonferroni threshold (72 tests). Mountain region corresponds to all states evaluated as a meta-analysis.

In a further evaluation of the altitude effect, we performed subsequent modeling that simultaneously considered the effect of altitude along with comorbidities and demographic factors was conducted. The outcomes for these models are presented in Fig. 3 and are adjusted for population density. When simultaneously evaluating the effects of several factors known to have an association with the dependent variable, the expectation is that they will remain significant when their contribution is independent of each other or that they will drop out when they share their covariance. In current models, the effect of altitude is consistently present when evaluating the data from the states of Colorado, Idaho, Wyoming and the whole Mountain region. The state of Colorado and the Mountain region were the only ones that consistently reached Bonferroni significance. Interestingly, using this set of models, the state of New Mexico displayed a reverse altitude effect where higher altitude was associated to a higher fatality rate. It is noteworthy to mention that in the states of Colorado, Idaho, and Wyoming, the predominant effect of comorbidities was eliminated suggesting that altitude is a more reliable predictor in these states. The states of Arizona, New Mexico, and Utah continued to display an association to comorbidities. Income continued to appear for several states and remained significant in Utah for the whole Mountain region. The higher risk of fatality due to COVID-19 observed for the whole Mountain region for Native Americans continues to be observed even when considering altitude although this effect was not significant at a Bonferroni level.

**Figure 4:**
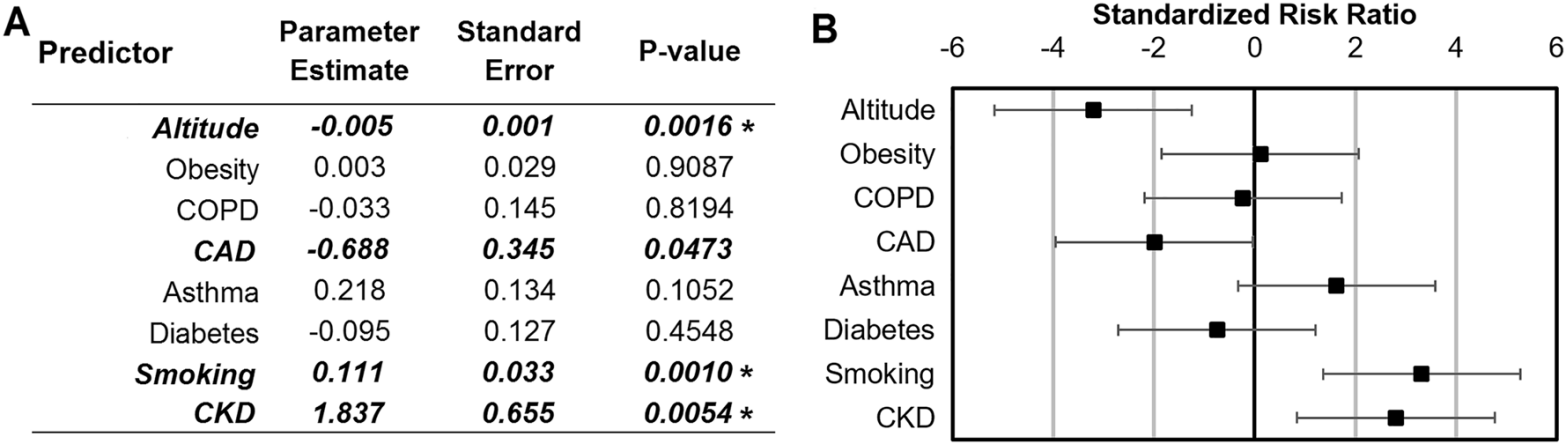
Elevation and comorbidities parameter risk ratio effect estimates for the mountain region adjusted by population density. (**A**) Generalized Additive Model parameter risk ratio estimates. (**B**) Standardized risk ratio estimates and confidence intervals. Values in bold are significant at P ≤ 0.05. Asterisk (*) indicates significance at a Bonferroni threshold (8 tests).

Based on the consistent detection of associations between comorbidities even while simultaneously considering the effect of altitude for the Mountain region, an all-inclusive model that included all comorbidities and altitude simultaneously and was adjusted for population density was developed. This model allows the detection and ranking of effects for predicting COVID-19 fatality rate. The outcome of this model is presented in Fig. 4, where raw parameter estimates are presented along with standardized effect ratios. In this model, comorbidities such as obesity, COPD, Asthma and Diabetes drop out in significance while only the effect of altitude and smoking continue to be significantly associated at a Bonferroni level. The standardized evaluation of these effects shows that altitude has a standardized effect of − 2.94, which is comparable in magnitude to the effects of smoking and CKD (3.48 and 2.58 respectively), although in the opposite direction. CHD displayed a significant but opposite effect to other comorbidity factors suggesting that patients with this condition may be self-selecting to live at higher altitudes.

Our findings suggest that altitude is not only a strong predictor for COVID-19 fatality, but that it has a fatality lowering effect, in some cases, this effect is more relevant than well documented comorbidities. Our study also confirmed and provided further evidence that comorbidities and demographics are important predictors of COVID-19 fatality.

## Discussion

The main question of evaluating the effect of altitude on COVID-19 fatality rates was explored early in the pandemic with some studies finding significant associations^13,18,28–30^ and some studies finding no associations^18,20–22,31,32^. As the pandemic advances and we have more information we believe it is important to revisit this argument. Studies that found no effect for altitude had several caveats which limited their conclusions earlier in the pandemic, which can now be reassessed with the new information available. These caveats can be put in three categories: The first group consists of studies that presented an argument based on early pandemic data^13,20–22,30^. These early studies closed their fatality data collection between April and July of 2020, with these dates being just a couple months into the pandemic. During that time, the spread of the virus was not geographically uniform, therefore, the case fatality data gathered earlier in the pandemic may not be an accurate representation of the true effect of altitude. Even when the virus was discovered in December of 2019, it was not declared a pandemic until March of 2020. This was followed by the implementation of travel and restrictions and social distancing mandates which further slowed the geographic expansion of the virus^33^. The second group consists of articles that make a regional assessment based on a small range of altitudes^21,22,30,34^. Studies done in Brazil, Spain, and Italy do not consider a wide range of altitudes in their analysis, which diminishes the ability to evaluate the effect of altitude on case fatality rate. The countries in these studies have the majority of their major population centers at altitudes less than 1 km above sea level, not accurately representing high altitude for this analysis. This group also includes studies done in locations that can have non generalizable characteristics such as those assessments done at a single location at extreme altitude in Peru^32^. The third, and last group includes studies that do not consider any covariate adjustments within their methodology^18,19,30^. Sociodemographic factors are strong determinants of health outcomes, so it is important to consider these variables for an effective assessment. Overall, many of the reports published early on the pandemic, such as the studies discussed above, fall into more than one of these groups.

The study we present revisits the main question of the effect of altitude on case fatality and addresses the caveats identified in previous studies and addresses them within the methodology: it includes over a full year of data ending in May 2021 which provides a more extensive representation of risk across geographies. It also focuses on a region that has a large altitude range and includes several major population centers at altitudes much higher than the aforementioned publications. Furthermore, it considers sociodemographic covariates in the analysis. The US Mountain Region is a sub region recognized by the US Census Bureau as a large generalizable area with defined geographic characteristics that facilitate context, excluding the relatively flat regions of the US, which dilutes the effect and increases the power of this study.

In our results, Colorado, Idaho, and Wyoming all displayed a strong altitude effect in which higher altitude decreased COVID-19 fatality rate; Arizona, Montana, Nevada, and Utah did not display any effect; and New Mexico displayed an inverse effect. Population density may have been a source of this discrepancy, and for that reason all our models are adjusted to this effect. As evident in Table 1, Colorado, Idaho, and Wyoming have higher average county altitudes of (2.061 km, 1.301 km, 1.687 km, respectively) than both Arizona (1.068 km) and Montana (1.151 km)^24^. CO, ID, and WY have, on average, higher average county altitudes, this causes the predictive effect of altitude on case-fatality rate is more likely observed. Population distribution across altitude within each state is another factor that contributes to the differing effects between states. For example, Colorado has 47% of its population in Arapahoe, Denver, El Paso, and Jefferson counties, where average county altitudes range from 1.637 km (Denver County) to 1.942 (El Paso County). On the other hand, Arizona^24^ has 62% of its population in Maricopa County (altitude of 0.385 km), and 75.3% of Nevada’s population lives in Clark County (altitude of 0.655 km)^24^. With a larger percentage of the population living at lower altitude in Nevada and Arizona, it is less likely the effect of high altitude on case-fatality rate would be seen. Other contributions to the differences in effects between states is the size and number of counties in each state. States that had a strong altitude effect, such as Colorado and Idaho, are divided into more counties than states that did not have an effect, such as Arizona, Nevada, and Utah. This discrepancy in the number of counties and the population within them is also addressed with the population density adjustment performed in all models. Altitude seems to have less predictive value on COVID-19 case fatality in states with fewer counties since each county covers a larger range of altitudes. States with more counties, however, allow for a more granular look at the data as the average altitude of each county encompasses a smaller area.

In the state of New Mexico, altitude was shown to have an association with an increase in the fatality rate. An explanation for this is the large Native American population that resides in northwest New Mexico; the state of New Mexico has one of the highest Native American populations at just under 200,000^35^. Native Americans have been impacted disproportionately by the pandemic^36^, and since the Native American population in this state is predominantly distributed in the high-altitude counties, that may have flipped the direction of the effect. A visual presentation of this distribution is displayed in Supplementary Fig. 1. Native Americans were the only racial group within the Mountain West Region that demonstrated a higher fatality rate from COVID-19. This is seen in Fig. 3. Although, this was not observed every time, most likely due to power limitations. Furthermore, Native Americans have been shown to be more prone to developing chronic illnesses like COPD, hypertension, diabetes, chronic kidney disease, and coronary artery disease. These chronic diseases have been linked to an increase in the rate of COVID-19 fatality^36,37^. Research on certain genes in the Native American population that alter ACE2^38^ is ongoing, potentially providing another explanation for the higher rates of COVID-19 fatality in this population. While this is an explanation for the different COVID-19 fatality trends seen in New Mexico, more research is required to provide a strong association.

Overall, altitude was shown to consistently decrease in fatality from COVID-19 infection even when controlling for comorbidities known to be associated with an increase in COVID-19 fatality. While this trend was not seen in every state individually, it was seen as a trend for the Mountain region collectively.

Much speculation has been presented already describing the human body’s physiological responses to high altitude and how it would play a role in a COVID-19 infection^30^. At high altitude, the body is required to adjust primarily as a result of the decreased PO2^39^. High altitude also has a significant effect on the cardiovascular system, which could potentially influence the progression of the COVID-19 virus. This is because much of the acclimatization to higher altitudes relies on improving oxygen extraction and delivery in the tissues. Therefore, patients with pre-existing conditions have a higher risk of severe COVID-19 infections^40^. Cardiovascular diseases (CVD) have been linked to increased severity of COVID-19^41^. Interestingly, residents at higher altitudes often have lower rates of cardiovascular diseases^42,43^. With healthier cardiovascular systems overall, patients with COVID-19 may be able to better handle the cardiovascular impact of the virus, thereby decreasing case-fatality rate. Similar to CVD, studies have shown that altitude and obesity have a similar inverse relationship^44–47^. Obesity has demonstrated an adverse effect on COVID-19 outcomes^48^. With lower rates of obesity at higher altitudes, the case-fatality rate from COVID-19 infections is likely to be lower. Our study showed that although comorbidities are important predictors to COVID-19 fatalities, the effect of altitude is in some cases more prominent and carries a positive protective effect. An explanation for documented comorbidities appearing to be less relevant as compared to altitude may be related to the geographic delimitation of our study. The Mountain region has a significant representation of communities at high altitudes as compared to the rest of the United States where altitude variation is not as extreme. Also, states within this region have a lower prevalence of comorbidity factors^49^. These two factors may contribute to the detection of the protective effect of altitude and the diminished relevance of other comorbidities.

Our study has some inherent limitations related to the methodology used and the geography of the region. We did not challenge official values by recalculating or considering possible fatalities. Counties are not broken up uniformly by state and may contain wide altitude ranges. As explained in the discussion, some states such as Arizona have only 17 counties while others such as Colorado have 64 counties even though they have comparable total areas. Future research could break down the analysis by smaller geographic areas such as by zip code, in order to obtain a more granular look at the effect of altitude. In addition, future research should be directed towards establishing the physiological changes that occur at altitude and how those changes affect COVID-19 fatality.

In summary, the goal of our study was to revisit a question asked early in the pandemic but with more information that is now available. We evaluated possible correlations between altitude and COVID-19 case fatality rate in a geographic region that has a wide dynamic representation of altitudes, all while considering comorbidities and sociodemographic factors. Our statistical analysis demonstrated that COVID-19 case-fatality rate decreased with higher altitude in the Mountain region, and this effect is evident even when controlling for pre-existing conditions and certain demographic variables. The protective effects of high altitude are likely influenced by both physiologic changes and demographic trends that are associated with life at high altitude. This effect is evident even when accounting for several variables known to impact COVID-19 case fatality rate; our study shows that case-fatality rate and altitude have an inverse relationship across the Mountain West Region. It is not realistic to make a broad recommendation to vulnerable populations to move to altitude to be better protected against poor COVID-19 outcomes. Nonetheless, our study highlights specific populations that are still vulnerable despite this extra protective factor. Allocation of resources to these specific population can improve overall outcomes.

## Supplementary Information

Supplementary Figure 1.

Supplementary Information 1.

Supplementary Information 2.

Supplementary Information 3.

Supplementary Information 4.

Supplementary Table 1.

## Data Availability

All data generated or analysed during this study are included in this published article in its supplementary information files.

## References

[1] Dong E, Du H, Gardner L (2020). An interactive web-based dashboard to track COVID-19 in real time. Lancet Infect. Dis..

[2] Onder G, Rezza G, Brusaferro S (2020). Case-fatality rate and characteristics of patients dying in relation to COVID-19 in Italy. JAMA.

[3] Hawkins RB, Charles EJ, Mehaffey JH (2020). Socio-economic status and COVID-19-related cases and fatalities. Public Health.

[4] Oshinubi K, Rachdi M, Demongeot J (2021). Analysis of reproduction number R0 of COVID-19 using current health expenditure as gross domestic product percentage (CHE/GDP) across countries. Healthcare.

[5] Kong JD, Tekwa EW, Gignoux-Wolfsohn SA (2021). Social, economic, and environmental factors influencing the basic reproduction number of COVID-19 across countries. PLoS One.

[6] Stephens KE, Chernyavskiy P, Bruns DR (2021). Impact of altitude on COVID-19 infection and death in the United States: A modeling and observational study. PLoS One.

[7] Srivastava S, Garg I, Bansal A, Kumar B (2020). SARS-CoV-2 infection: Physiological and environmental gift factors at high altitude. Virus Dis..

[8] Arias-Reyes C (2020). Does the pathogenesis of SARS-CoV-2 virus decrease at high-altitude?. Respir. Physiol. Neurobiol..

[9] Burtscher M (2014). Effects of living at higher altitudes on mortality: A narrative review. Aging Dis..

[10] Horner A (2017). Altitude and COPD prevalence: Analysis of the PREPOCOL-PLATINO-BOLD-EPI-SCAN study. Respir. Res..

[11] Simeonov KP, Himmelstein DS (2015). Lung cancer incidence decreases with elevation: Evidence for oxygen as an inhaled carcinogen. PeerJ.

[12] Demongeot J, Flet-Berliac Y, Seligmann H (2020). Temperature decreases spread parameters of the new Covid-19 case dynamics. Biology (Basel).

[13] Demongeot, J. *et al.* The application of ARIMA model to analyze COVID-19 incidence pattern in several countries. *J. Math. Comput. Sci.* **12** (2022).

[14] Seligmann H, Vuillerme N, Demongeot J (2021). Unpredictable, counter-intuitive geoclimatic and demographic correlations of COVID-19 spread rates. Biology (Basel).

[15] Seligmann H, Iggui S, Rachdi M, Vuillerme N, Demongeot J (2020). Inverted covariate effects for first versus mutated second wave Covid-19: High temperature spread biased for young. Biology (Basel).

[16] Khodaee M, Grothe HL, Seyfert JH, VanBaak K (2016). Athletes at high altitude. Sports Health.

[17] Liu M (2020). Potential role of ACE2 in coronavirus disease 2019 (COVID-19) prevention and management. J. Transl. Intern. Med..

[18] Segovia-Juarez J, Castagnetto JM, Gonzales GF (2020). High altitude reduces infection rate of COVID-19 but not case-fatality rate. Respir. Physiol. Neurobiol..

[19] Valverde-Bruffau VJ, Cárdenas L, Gonzales GF (2020). The pathogenicity of COVID-19 is independent of increasing altitude: The case of Colombia. Am. J. Trop. Med. Hyg..

[20] Woolcott OO, Bergman RN (2020). Mortality attributed to COVID-19 in high-altitude populations. High Alt. Med. Biol..

[21] Castilla J, Fresán U, Trobajo-Sanmartín C, Guevara M (2021). Altitude and SARS-CoV-2 infection in the first pandemic wave in Spain. Int. J. Environ. Res. Public Health.

[22] Perone G (2021). The determinants of COVID-19 case fatality rate (CFR) in the Italian regions and provinces: An analysis of environmental, demographic, and healthcare factors. Sci. Total Environ..

[23] US Center for Disease Control. 2020 PLACES. Local data for better health county data 2020 release. https://chronicdata.cdc.gov/500-Cities-Places/PLACES-Local-Data-for-Better-Health-County-Data-20/swc5-untb/data (2021).

[24] US Census Bureau. 2019 American community survey 5-year public use microdata samples. https://data.census.gov/cedsci/ (2020).

[25] Hastie T, Tibshirani R (1995). Generalized additive models for medical research. Stat. Methods Med. Res..

[26] Hastie, T. J. Generalized additive models. in *Statistical Models in S* 249–307 (Routledge, 2017).

[27] Ravindra K, Rattan P, Mor S, Aggarwal AN (2019). Generalized additive models: Building evidence of air pollution, climate change and human health. Environ. Int..

[28] Cano-Pérez E (2020). Negative correlation between altitude and COVID-19 pandemic in Colombia: A preliminary report. Am. J. Trop. Med. Hyg..

[29] Quevedo-Ramirez A, Al-kassab-Córdova A, Mendez-Guerra C, Cornejo-Venegas G, Alva-Chavez KP (2020). Altitude and excess mortality during COVID-19 pandemic in Peru. Respir. Physiol. Neurobiol..

[30] Fernandes JSC (2021). Altitude conditions seem to determine the evolution of COVID-19 in Brazil. Sci. Rep..

[31] Cardenas L, Valverde-Bruffau V, Gonzales GF (2021). Altitude does not protect against SARS-CoV-2 infections and mortality due to COVID-19. Physiol. Rep..

[32] Champigneulle B (2021). High-altitude environment and COVID-19: SARS-CoV-2 seropositivity in the highest city in the world. High Alt. Med. Biol..

[33] Stawicki SP (2020). The 2019–2020 novel coronavirus (severe acute respiratory syndrome coronavirus 2) pandemic: A Joint American College of Academic International Medicine-World Academic Council of Emergency Medicine Multidisciplinary COVID-19 Working Group Consensus Paper. J. Glob. Infect. Dis..

[34] Cascetta E, Henke I, Di Francesco L (2021). The effects of air pollution, sea exposure and altitude on COVID-19 hospitalization rates in Italy. Int. J. Environ. Res. Public Health.

[35] US Census Bureau. QuickFacts New Mexico. https://www.census.gov/quickfacts/NM (2020).

[36] Carethers JM (2021). Insights into disparities observed with COVID-19. J. Intern. Med..

[37] Abuelgasim E, Saw LJ, Shirke M, Zeinah M, Harky A (2020). COVID-19: Unique public health issues facing Black, Asian and minority ethnic communities. Curr. Probl. Cardiol..

[38] Khayat AS (2020). ACE2 polymorphisms as potential players in COVID-19 outcome. PLoS One.

[39] Constanzo L (2017). Physiology.

[40] Zheng Y-Y, Ma Y-T, Zhang J-Y, Xie X (2020). COVID-19 and the cardiovascular system. Nat. Rev. Cardiol..

[41] Yang J (2020). Prevalence of comorbidities and its effects in patients infected with SARS-CoV-2: A systematic review and meta-analysis. Int. J. Infect. Dis..

[42] Faeh D, Gutzwiller F, Bopp M, Swiss National Cohort Study Group (2009). Lower mortality from coronary heart disease and stroke at higher altitudes in Switzerland. Circulation.

[43] Savla JJ, Levine BD, Sadek HA (2018). The effect of hypoxia on cardiovascular disease: Friend or foe?. High Alt. Med. Biol..

[44] Merrill RM (2020). Explaining the inverse association between altitude and obesity. J. Obes..

[45] Voss JD, Allison DB, Webber BJ, Otto JL, Clark LL (2014). Lower obesity rate during residence at high altitude among a military population with frequent migration: A quasi experimental model for investigating spatial causation. PLoS One.

[46] Voss JD, Masuoka P, Webber BJ, Scher AI, Atkinson RL (2013). Association of elevation, urbanization and ambient temperature with obesity prevalence in the United States. Int. J. Obes..

[47] Woolcott OO (2016). Inverse association between altitude and obesity: A prevalence study among andean and low-altitude adult individuals of Peru. Obesity.

[48] Petrakis D (2020). Obesity—A risk factor for increased COVID-19 prevalence, severity and lethality (Review). Mol. Med. Rep..

[49] United Health Foundation. *Anual Report 2020*. https://assets.americashealthrankings.org/app/uploads/annual20-rev-complete.pdf (2020).

