# Supplementary material for: Revisiting the COVID-19 fatality rate and altitude association through a comprehensive analysis
Source: Sci Rep. 2022 Oct 27;12:18048. doi: 10.1038/s41598-022-21787-z (PMC9610325; doi:10.1038/s41598-022-21787-z)
Supplement: Supplementary file 1 — Supplementary Figure 1. [file 41598_2022_21787_MOESM1_ESM.pdf]

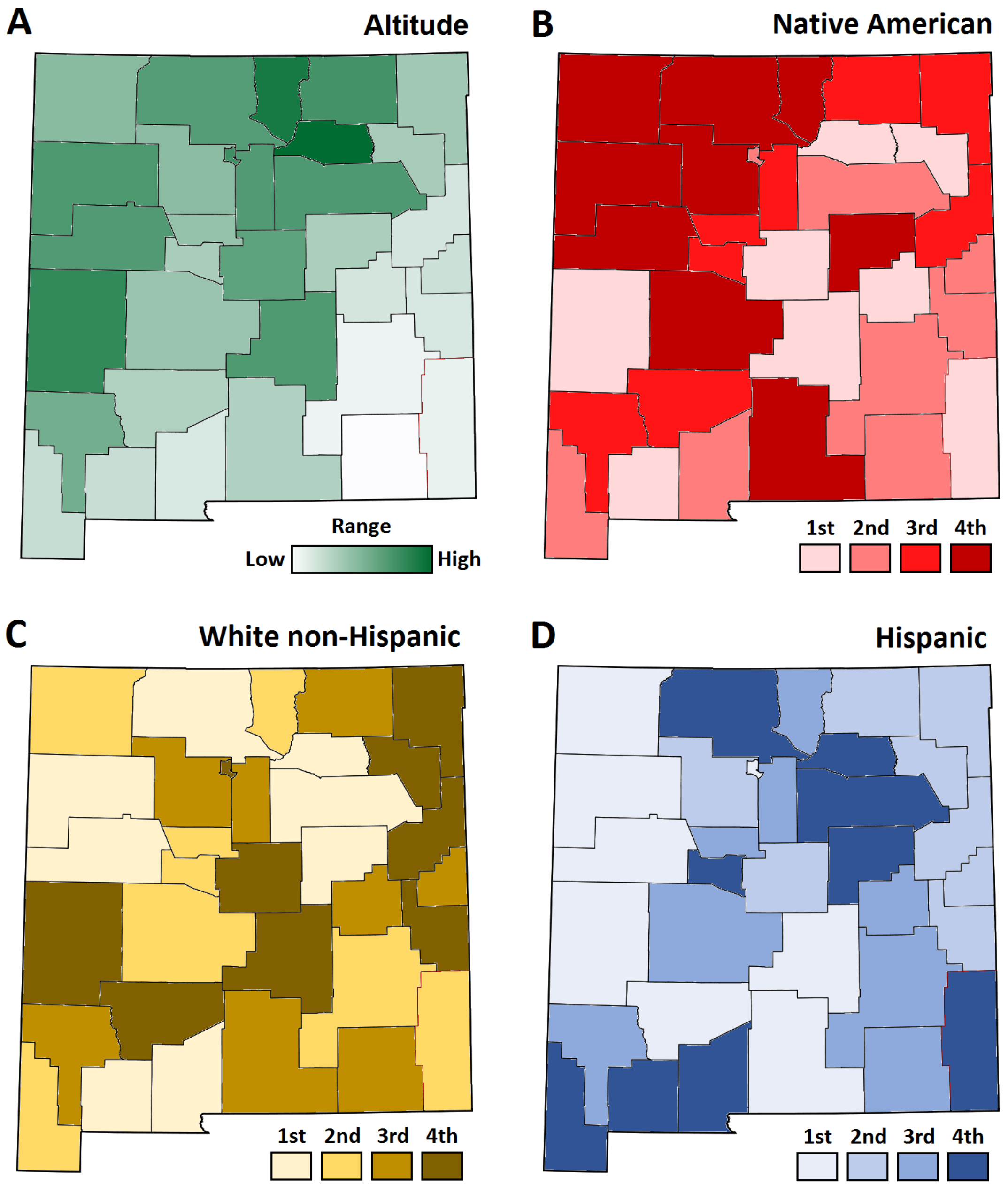

Supplementary Figure 1. Visual comparison between county altitude and three major population types in the state of New Mexico. A) Population weighted mean average altitude by county. Darker shader corresponds to higher altitude B) Quartile proportion of the Native American population by county. C) Quartile proportion of the White non-Hispanic population by county. D) Quartile proportion of the Hispanic population by county. Darker shaders in B, C and D correspond to higher proportion of such ethnicity in the given county.
