# Supplementary material for: Revisiting the COVID-19 fatality rate and altitude association through a comprehensive analysis
Source: Sci Rep. 2022 Oct 27;12:18048. doi: 10.1038/s41598-022-21787-z (PMC9610325; doi:10.1038/s41598-022-21787-z)
Supplement: Supplementary file 2 — Supplementary Information 1. [file 41598_2022_21787_MOESM2_ESM.pdf]

Official State's COVID-19 data resource webpages

As of July 23, 2021

Arizona

ADHS – COVID-19 Deaths
https://www.azdhs.gov/covid19/data/index.php#deaths

Colorado

COVID-19 data | Colorado COVID 19 Updates
https://covid19.colorado.gov/data

Idaho

DPH Idaho COVID-19 Dashboard | Tableau Public
https://public.tableau.com/app/profile/idaho.division.of.public.health/viz/DPHIdahoCOVID-19Dashboard/Home

Montana

COVID 19 Home
https://covid19.mt.gov/

Nevada

Coronavirus (COVID-19) in Nevada | Nevada Health Response
https://nvhealthresponse.nv.gov/

New Mexico

Epidemiology Reports | NMDOH – Coronavirus Updates
https://cv.nmhealth.org/epidemiology-reports/

Utah

Case Counts | coronavirus
https://coronavirus.utah.gov/case-counts/

Wyoming

Coronavirus Disease 2019 (COVID-19) Wyoming Department of Health
https://health.wyo.gov/publichealth/infectious-disease-epidemiology-unit/disease/novel-coronavirus/
