# Supplementary material for: Revisiting the COVID-19 fatality rate and altitude association through a comprehensive analysis
Source: Sci Rep. 2022 Oct 27;12:18048. doi: 10.1038/s41598-022-21787-z (PMC9610325; doi:10.1038/s41598-022-21787-z)
Supplement: Supplementary file 6 — Supplementary Table 1. [file 41598_2022_21787_MOESM6_ESM.pdf]

Supplementary Table 1. Mean and standard deviations for county comorbidities, prevalence and demographic factors by state and by mountain region.

	Arizona		Colorado		Idaho		Montana		New Mexico		Nevada		Utah		Wyoming		Mountain Region	
	Mean	Std Dev	Mean	Std Dev	Mean	Std Dev	Mean	Std Dev	Mean	Std Dev	Mean	Std Dev	Mean	Std Dev	Mean	Std Dev	Mean	Std Dev
Average county comorbidity prevalence by state (percentage)																		
Obesity	0.3287	0.0372	0.2526	0.0367	0.3189	0.0286	0.3117	0.0361	0.3310	0.0405	0.3187	0.0287	0.3116	0.0401	0.3000	0.0335	0.3020	0.0450
COPD	0.0752	0.0156	0.0552	0.0117	0.0670	0.0073	0.0643	0.0110	0.0658	0.0131	0.0766	0.0085	0.0550	0.0091	0.0646	0.0068	0.0632	0.0124
CAD	0.0695	0.0104	0.0556	0.0078	0.0647	0.0056	0.0624	0.0085	0.0660	0.0093	0.0676	0.0061	0.0564	0.0071	0.0585	0.0044	0.0614	0.0088
Asthma	0.1097	0.0150	0.0948	0.0048	0.0976	0.0042	0.0989	0.0096	0.1008	0.0111	0.0927	0.0052	0.0973	0.0072	0.0916	0.0050	0.0974	0.0087
Diabetes	0.1091	0.0197	0.0801	0.0155	0.1014	0.0111	0.0916	0.0174	0.1178	0.0203	0.0996	0.0109	0.0931	0.0127	0.0841	0.0081	0.0945	0.0192
Smoking	0.1936	0.0331	0.1753	0.0284	0.1882	0.0193	0.2116	0.0343	0.1879	0.0328	0.2059	0.0227	0.1227	0.0239	0.1935	0.0210	0.1849	0.0370
CKD	0.0329	0.0048	0.0265	0.0036	0.0284	0.0023	0.0271	0.0038	0.0325	0.0044	0.0282	0.0023	0.0265	0.0028	0.0254	0.0017	0.0280	0.0040
Age (number of people)																		
Under 18 years old	107,193	260,623	19,434	40,376	9,879	19,177	3,977	6,971	14,562	26,734	39,369	121,222	31,458	67,798	5,815	5,712	20,302	75,787
18 to 34 years old	104,512	257,567	20,801	45,453	8,448	17,149	3,946	7,854	14,032	28,744	39,167	121,053	26,953	62,088	5,530	5,986	19,677	75,254
35 to 64 years old	168,038	404,750	33,691	68,155	14,075	29,060	7,063	12,279	22,995	45,513	67,173	203,709	35,347	80,734	9,432	9,001	31,439	118,136
65 years old and over	79,672	161,647	11,850	22,329	5,923	10,742	3,321	5,251	10,532	18,994	26,637	75,693	11,374	23,666	3,872	3,398	12,880	46,384
Sex (number of people)																		
Male	226,282	534,432	42,902	87,323	19,133	37,533	9,186	16,045	30,516	58,436	86,016	259,357	52,763	116,823	12,516	11,892	41,934	155,586
Female	233,133	549,128	42,873	87,796	19,192	38,242	9,122	16,015	31,605	61,172	86,330	262,250	52,370	116,053	12,132	11,769	42,365	158,976
Race (number of people)																		
White	355,757	843,041	72,124	141,690	34,499	67,876	16,249	29,579	46,583	90,344	113,273	316,356	90,956	191,941	22,578	21,538	67,616	238,451
Black	20,165	60,958	3,481	12,767	254	917	86	217	1,244	3,414	15,420	60,077	1,224	3,990	223	432	3,150	21,475
Native American	20,554	25,435	811	1,600	503	774	1,136	2,219	5,944	13,544	2,181	4,463	1,132	2,108	586	1,656	2,582	8,818
Asian	15,269	45,998	2,732	7,202	542	1,758	142	369	969	3,062	14,170	51,151	2,431	8,477	211	327	2,790	17,178
Pacific Islander	889	2,430	130	402	60	147	14	36	49	116	1,156	3,925	936	3,110	25	53	264	1,528
Other	29,763	73,590	3,346	8,706	1,340	3,705	121	255	5,318	12,732	17,682	59,594	5,283	19,189	370	507	4,855	24,497
Mixed	17,018	40,420	3,151	7,653	1,126	2,605	559	1,044	2,015	4,876	8,465	27,841	3,171	7,797	657	789	3,044	12,803
Hispanic	143,785	340,648	18,447	42,915	4,783	9,746	694	1,425	30,300	61,542	49,519	162,373	14,730	39,845	2,420	3,158	21,036	96,723
White (Non Hispanic)	252,176	599,131	58,506	115,181	31,460	63,149	15,807	28,695	23,292	45,977	84,988	226,057	82,444	172,514	20,765	19,258	52,948	175,206
Education (number of people)																		
Less than high school	39,074	87,639	4,772	11,249	2,282	3,680	795	1,172	5,899	9,671	15,676	49,166	4,748	12,152	1,112	1,122	5,911	25,551
High school completed	73,585	160,582	12,346	24,267	6,781	12,035	3,636	5,926	11,042	18,991	33,275	100,819	14,139	31,103	4,828	4,484	13,691	49,216
Some college or associate's	104,941	235,728	17,426	34,813	8,983	17,668	4,176	7,010	13,322	25,687	40,280	118,314	22,047	47,553	6,147	6,244	18,589	68,664
Bachelor's or higher	92,553	240,502	24,366	53,835	6,969	18,334	4,121	8,404	11,584	28,561	29,663	89,025	21,307	51,681	4,624	4,698	18,135	69,413
Employment (number of people)																		
Employed	207,737	524,725	45,201	94,049	17,940	37,747	9,093	17,070	26,848	56,504	82,685	250,468	51,274	119,289	12,475	12,040	40,473	152,984
Unemployed	12,837	27,734	2,014	4,178	803	1,620	374	617	1,901	3,500	5,430	17,074	1,889	4,309	573	625	2,138	8,410
Poverty level (number of people)																		
Poverty under 50% FPL	32,372	69,313	4,059	8,484	2,048	3,868	1,028	1,602	5,160	9,174	10,315	32,872	4,556	9,908	1,186	1,156	4,975	19,705
Poverty under 125% FPL	91,517	195,382	11,833	24,116	6,839	11,529	3,279	5,119	15,420	27,197	30,604	95,337	14,181	30,101	3,585	3,369	14,724	56,035
Poverty under 150% FPL	113,150	240,942	14,839	30,196	8,822	14,777	4,088	6,467	18,887	33,111	38,553	120,350	18,340	38,354	4,541	4,214	18,431	69,503
Poverty under 185% FPL	145,055	312,008	19,648	39,862	11,799	19,678	5,366	8,649	23,421	41,111	50,433	156,343	25,152	52,729	6,059	5,614	24,028	90,116
Poverty under 200% FPL	158,524	341,445	21,802	44,241	13,128	22,175	5,931	9,562	25,337	44,508	55,688	173,216	28,107	58,953	6,718	6,218	26,460	98,947
Poverty under 300% FPL	239,291	526,807	35,151	71,061	20,585	35,594	9,241	15,237	35,868	63,824	87,582	271,527	49,024	102,944	11,003	10,127	41,315	153,609
Poverty under 400% FPL	302,680	677,882	47,189	95,460	26,470	47,224	11,925	19,975	44,040	80,185	113,461	349,772	66,783	141,585	14,870	13,851	53,572	198,389
Poverty under 500% FPL	348,403	788,258	56,885	115,199	30,348	55,487	13,961	23,725	49,709	91,821	131,555	402,896	79,308	170,258	17,915	16,793	62,536	230,907
Income (Dollars)																		
Families median	$58,103	$11,006	$73,195	$20,321	$61,845	$ 9,151	$64,868	$10,958	$55,867	$19,419	$72,819	$12,189	$73,694	$15,121	$76,856	$11,811	$67,246	$16,375
Non family median	$28,867	$ 7,137	$36,436	$11,792	$28,837	$ 4,506	$29,989	$ 5,253	$27,154	$11,323	$36,052	$ 7,315	$33,669	$ 8,810	$35,964	$ 9,277	$32,113	$ 9,360
